# New oral spherical carbon adsorbent effectively reduces serum indoxyl sulfate levels in moderate to advanced chronic kidney disease patients: a multicenter, prospective, open-label study

**DOI:** 10.1186/s12882-020-01971-x

**Published:** 2020-07-31

**Authors:** Seok-hyung Kim, Jong Hyun Jhee, Hoon Young Choi, Sang-Ho Lee, Sug Kyun Shin, So-Young Lee, Dong Ho Yang, Joo-Hark Yi, Sang-Woong Han, Young-Il Jo, Hyeong Cheon Park

**Affiliations:** 1 Department of Internal Medicine, Gangnam Severance Hospital, Yonsei University College of Medicine, 211 Eonju-ro, Gangnam-gu, Seoul, 06273 South Korea; 2 Division of Nephrology, Department of Internal Medicine, Hallym University Chuncheon Sacred Heart Hospital, Hallym University College of Medicine, Chuncheon, South Korea; 3 Severance Institute for Vascular and Metabolic Research, Yonsei University College of Medicine, Seoul, 03722 South Korea; 4 Division of Nephrology, Department of Internal Medicine, Kyunghee University Hospital at Gangdong, College of Medicine, Kyunghee University, Seoul, South Korea; 5 Department of Internal Medicine, National Health Insurance Service Medical Center, Ilsan Hospital, Goyang-si, Gyeonggi-do South Korea; 6 Division of Nephrology, Department of Internal Medicine, CHA Bundang Medical Center, CHA University, Pocheon-si, Gyeongi-do South Korea; 7 Division of Nephrology, Department of Internal Medicine, Hanyang University Guri Hospital, Guri, Gyeonggi-do South Korea; 8 Division of Nephrology, Department of Internal Medicine, Konkuk University Medical Center, Seoul, South Korea

**Keywords:** Chronic kidney disease, Indoxyl sulfate, Carbon adsorbent, Oral spherical carbon adsorbent, Uremic toxin

## Abstract

**Background:**

Elevated levels of serum indoxyl sulfate (IS) have been linked to cardiovascular complications in patients with chronic kidney disease (CKD). Oral sorbent therapy using spherical carbons selectively attenuates IS accumulation in CKD patients. This study aimed to investigate whether oral administration of a new oral spherical carbon adsorbent (OSCA), reduces serum IS levels in moderate to severe CKD patients.

**Methods:**

This prospective, multicenter, open-label study enrolled patients with CKD stages 3–5. Patients were prescribed OSCA for 8 weeks (6 g daily in 3 doses) in addition to standard management. Serum IS levels were measured at baseline and 4 and 8 weeks of treatment with OSCA.

**Results:**

A total of 118 patients were enrolled and 87 eligible patients completed 8 weeks of study. The mean age of the study subjects was 62.8 ± 13.7 years, and 80.5% were male. Baseline levels of serum IS were negatively correlated with estimated glomerular filtration rate (eGFR) (r = − 0.406, *P* < 0.001) and increased with increasing CKD stages (stage 3, 0.21 ± 0.21 mg/dL; stage 4, 0.54 ± 0.52 mg/dL; stage 5, 1.15 ± 054 mg/dL; *P* for trend *=* 0.001). The patients showed significant reduction in serum total IS levels as early as 4 weeks after OSCA treatment (22.5 ± 13.9% reduction from baseline, *P* < 0.001) and up to 8 weeks (31.9 ± 33.7% reduction from baseline, *P* < 0.001). This reduction effect was noted regardless of age, kidney function, or diabetes. No severe adverse effects were reported. Gastrointestinal symptoms were the most commonly reported adverse effects. In total, 21 patients withdrew from the study, with dyspepsia due to heavy pill burden as the most common reason. The medication compliance rate was 84.7 ± 21.2% (min 9%, max 101%) for 8 weeks among those who completed the study.

**Conclusions:**

OSCA effectively reduced serum IS levels in moderate to severe CKD patients. Gastrointestinal symptoms were the most commonly reported complications, but no treatment-related severe adverse effects were reported.

**Trial registration:**

Clinical Research Information Service (KCT0001875. 14 December 2015.)

## Background

Patients with chronic kidney disease (CKD) have higher rates of cardiovascular (CV) morbidity and mortality than the general population [1–3]. Non-traditional risk factors, including increased inflammation, endothelial dysfunction, vascular calcification, and uremic toxins, that are relevant to patients with CKD may result in increased CV events [4–8]. Indoxyl sulfate (IS), a protein-bound uremic toxin, is a dietary tryptophan metabolite [9, 10]. Ingested tryptophan is converted to indole by intestinal microbiota, absorbed into the intestine, and converted into IS by the liver [11]. IS is eventually excreted into the urine via proximal tubular secretion. However, in patients with CKD, decreased renal clearance leads to an increase in blood and tissue levels of IS [12]. Increased IS levels are associated with renal inflammatory gene activation and increased expression of profibrotic genes and proteins leading to renal tubulointerstitial fibrosis [13–17]. These findings suggest that high levels of IS act as nephrotoxins and are responsible for the progression of CKD.

Such toxicity to the kidney has prompted the development of therapeutic strategies to reduce the levels of IS in circulation through dialytic and non-dialytic treatments [18]. As IS is derived from colonic microbiota metabolism, there have been efforts to target the intestine to reduce its production [11]. This has been done particularly through maintaining normal gut homeostasis by modulating bacterial growth in the colon, thus reducing bacterial toxin production, or inhibiting gastrointestinal absorption of protein-bound uremic toxin precursors by adsorbing and enhancing their excretion into the feces [19]. Compared to conventional renal replacement therapy, both treatment strategies offer benefits in terms of lower costs, fewer complications, and merits of application in non-dialysis CKD patients.

AST-120, a synthesized carbon adsorbent, has been used in Asian countries to treat progressive CKD patients for the last 10–20 years. This oral sorbent selectively adsorbs indole in the lower intestinal tract and thereby attenuates the systemic accumulation of IS in CKD patients. Benefits of oral absorbents have been demonstrated in preclinical and non-randomized clinical studies to reduce serum levels of IS by 11.3 to 39% and improve clinical outcomes [14, 20–25]. However, many patients report gastrointestinal complications and difficulties in ingesting AST-120 in practice. Of note, the difficulties in ingesting a large amount of medications and their adverse effect on medication compliance have been highlighted. Recently, a new orally administered carbon adsorbent capsule was developed in Korea (Renamezin®, Daewon Pharm; Seoul, Korea) (Additional file 1, Fig. S1A and 1B). In contrast to other oral absorbents, this new oral spherical carbon adsorbent (OSCA) uses furan resin as the raw material and is reported to have 2 to 10 times greater compression strength when compared with spherical carbonaceous adsorbents made from petroleum pitch_._ However, there are no clinical data regarding the efficacy of OSCA in reducing IS levels in moderate to severe CKD patients.

The aim of this study was to investigate whether a newly developed oral adsorbent, OSCA, effectively reduces serum levels of IS in moderate to severe CKD patients. In particular, the ability of OSCA to show comparable benefits in CKD patients with diabetes as well as in those without was evaluated. Furthermore, we aimed to investigate the adverse effects and medication compliance associated with OSCA.

## Methods

### Patient selection and study design

Moderate to severe CKD patients not on dialysis treatment from six outpatient nephrology clinics in Korea were enrolled for this prospective, open-label, observation study. Eligible patients were those 1) who provided informed consent, 2) over 19 years of age, 3) with serum creatinine levels of 1.5–5.0 mg/dL, and 4) who showed no spontaneous improvement in or progression of renal disease or any changes in their conventional treatments for at least 3 months prior to the study. This study excluded patients with 1) a colonic transit disorder; 2) recurrent constipation; 3) peptic ulcer or gastroesophageal varices; 4) uncontrolled hypertension; 5) cardiovascular disease (coronary artery disease, myocardial ischemia, cerebrovascular disease, or peripheral artery disease) in the 6 months prior to enrollment; 6) uncontrolled arrhythmia; 7) liver disease with serum aminotransferase levels greater than 2 times the upper normal limit; 8) history of alcohol abuse; and 9) active infection or inflammation, and patients who were 10) pregnant, lactating, or in the reproductive age desiring pregnancy or 11) those determined to be inappropriate for clinical study by the investigators.

The study protocol was approved by each site’s institutional review board (3–2015-0271, ClinicalTrials.gov Identifier: NCT02681991) and performed in accordance with the ethical principles of the Declaration of Helsinki. All patients provided written informed consent before participation in the study.

### Data collection

All eligible patients’ medical charts were carefully reviewed to identify any concomitant mediations and comorbidities. Standard management for CKD was provided to all study participants as follows: dietary education on low-salt and low-protein diets (daily sodium less than 2.3 g and dietary protein of 0.6–0.8 g, respectively) was provided to all participants at outpatient clinic visits [26]. Hypertension was controlled with antihypertensive medications, including angiotensin-converting enzyme inhibitors (ACEIs) and/or angiotensin II receptor blockers (ARBs), calcium channel blockers, and diuretics as needed. The new carbon adsorbent medication, OSCA, was kindly provided by Daewon Pharmaceutical. The dose of OSCA used in this study was the dose recommended to obtain renoprotective effects in progressive CKD patients, whose serum creatinine concentrations were between 2.0 and 5.0 mg/dL, by the Korean Ministry of Food and Drug Safety. Patients were instructed to take 6.0 g/day of OSCA (Renamezin®, a total of 21 capsules daily) in 3 divided doses for 8 weeks in addition to their standard management. In order to investigate medication compliance rates to mimic the outpatient clinic environment, patients were encouraged to report any adverse events experienced during the study period. Furthermore, the primary investigators of each institute were advised to discontinue this regime for any individuals experiencing adverse events with more than mild severity. The medication compliance was assessed based on meticulous record-keeping of the number of pills in the medication bottles returned by each individual patient.

Patients were followed up prospectively at baseline and at 4 and 8 weeks of treatment. At each visit, clinical parameters, such as blood pressure, pulse rates, and body weight, and laboratory parameters, including complete blood cell count and serum phosphorus, serum uric acid, blood urea nitrogen (BUN), serum creatinine, serum albumin, serum electrolyte, and serum IS levels, were measured. The estimated glomerular filtration rate (eGFR) was calculated using the Modification of Diet in Renal Disease Study (MDRD) equation [27]. Urine samples were collected in the morning after the first voiding, and the urine protein to creatinine ratio (UPCR) were calculated to assess proteinuria.

### Measurement of serum indoxyl sulfate levels

Serum total IS levels were measured using a high-performance liquid chromatography-fluorescence detector (HPLC-FLD, Agilent 1100 series; Agilent Technologies, Santa Clara, CA) at an independent central lab (Seoul Clinical Laboratories, Seoul, Korea). Participants’ serum samples (300 μL) were dispensed into 0.22-μm nylon centrifugal filter tubes (F2517–1, National Scientific; Claremont, CA) and centrifuged at 13,000 rpm for 5 min. The upper layers (200 μL) were collected into HPLC vials, and 10 μl was injected into the HPLC-FLD using an HPLC auto-sampler. A guard column (CAPCELLPAK MF, SG80 5 m, 4.6 mm ID X 150 mm, 40.0 °C; Shiseido, Tokyo, Japan) was used. Eight standard samples of IS at concentrations from 0 to 10.0 mg/dL were prepared for calibration. The flow rate was 1.0 mL/min, and the total run time was 11 min. Fluorescence intensities between 295 nm in excitation and 390 nm in emission were measured. The reference normal range of serum IS derived from 150 healthy controls without any underlying disease was 0.07 ± 0.04 mg/dL [28].

### Sample size and statistical analysis

To estimate sample size, we used results from previous clinical trials on oral adsorbents in CKD and dialysis patients [20–25]. Assuming a 25 to 30% reduction effect of previous oral adsorbents on serum levels of IS, at least 50 patients were needed (α < 0.05 and power of 80%). Sample size calculations were performed using the general power analysis program (G*Power, http:// gpower.hhu.de/) [29]. Post-marketing data on oral adsorbents showed an up to 50% dropout rate. Therefore, a sample size of 120 patients was initially needed for the study. Statistical differences in the clinical characteristics before and after treatment with OSCA were determined using Student’s t-test for continuous variables. A repeated measures analysis of variance test was used to determine the reduction of serum IS levels at 4 and 8 weeks of treatment. The chi-squared test was used to assess the degree of medication compliance and reduction of serum sulfate levels. Pearson’s correlation coefficients were used to assess the relationships between serum IS levels and selected clinical or biochemical variables. Multiple linear regression analysis was performed to identify factors independently associated with serum IS. All analyses were performed using SPSS 23 software for Windows (IBM; Armonk, NY), and data are presented as mean ± standard deviation (SD) for continuous variables or frequency (percentage) for categorical variables. *P*-values less than 0.05 were considered to be statistically significant.

## Results

### Baseline characteristics

Table 1 shows the baseline demographic, clinical, and biochemical characteristics of the 87 analyzed patients. The mean age of the study participants was 62.8 ± 13.7 years, and 70 (80.5%) were male. The mean systolic and diastolic blood pressure were 131.2 ± 11.7 and 79.0 ± 11.2 mmHg. The mean duration of CKD among total study subjects were 70.0 ± 47.2 months and 95.4% of subjects were in CKD stage 3 or 4. The prevalence of hypertension was 77.0% and that of diabetes were 48.3%. In the laboratory tests at baseline, mean levels of IS were 0.46 ± 0.41 mg/dL and mean eGFR levels were 31.6 ± 11.3 mL/min/1.73m^2^ among study subjects.

**Table 1:** Baseline characteristics of study participants

	(***n*** = 87)
**Age (years)**	62.8 ± 13.7
**Sex, male (%)**	70 (80.5)
**BMI (kg/m**^**2**^**)**	25.9 ± 4.1
**Systolic BP (mmHg)**	131.2 ± 11.7
**Diastolic BP (mmHg)**	79.0 ± 11.2
**CKD duration (months)**	70.0 ± 47.2
**CKD stage**	
Stage 3 (%)	45 (51.7)
Stage 4 (%)	38 (43.7)
Stage 5 (%)	3 (3.4)
**Comorbidities (%)**	
Hypertension	67 (77.0)
Diabetes	41 (48.3)
Cardiovascular disease	13 (14.9)
Cerebrovascular disease	10 (11.5)
**Laboratory data**	
Indoxyl sulfate (mg/dL)	0.46 ± 0.41
BUN (mg/dL)	35.1 ± 12.7
Serum creatinine (mg/dL)	2.5 ± 0.8
eGFR (mL/min/1.73m^2^)	31.6 ± 11.3
Urine PCR (mg/g)	3613.4 ± 4203.2
Hemoglobin (g/dL)	12.1 ± 2.0
Phosphorus (mg/dL)	3.6 ± 0.6
Intact PTH (mg/dL)	104.0 ± 101.3
Uric acid (mg/dL)	7.1 ± 1.8
Albumin (g/dL)	3.9 ± 0.6
Serum Na (mmol/L)	139.5 ± 2.5
Serum K (mmol/L)	4.8 ± 0.6
Total CO_2_ (mmol/L)	23.8 ± 3.3

Data are presented as mean ± standard deviation or number (percentage) of subjects

***Abbreviation***: *BMI* body mass index, *BP* blood pressure, *CKD* chronic kidney disease, *eGFR* estimated glomerular filtration rate, *PCR* protein to creatinine ratio, *PTH* parathyroid hormone

### Association between serum indoxyl sulfate levels and clinical parameters

**Fig. 1:**
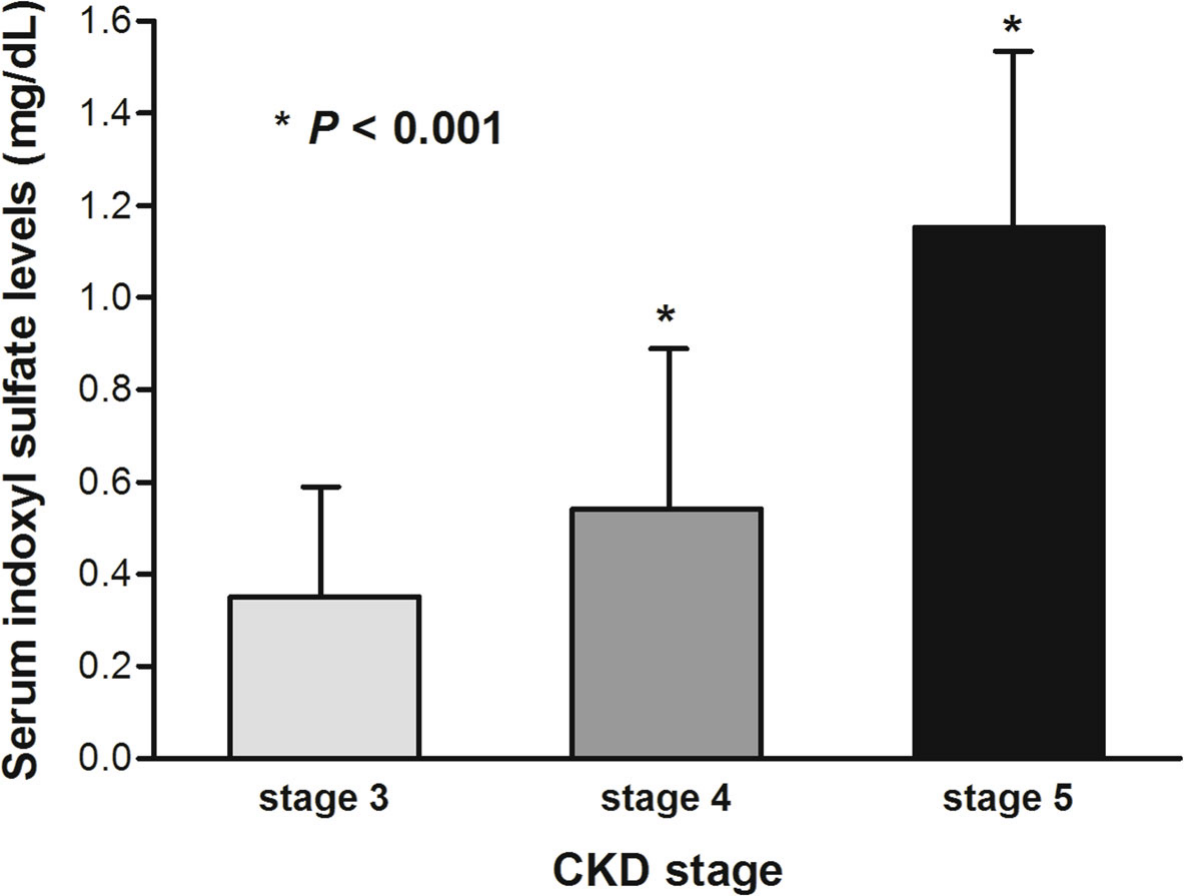
Serum levels of indoxyl sulfate presented as a function of CKD stages. *Abbreviation:* CKD, chronic kidney disease

**Fig. 2:**
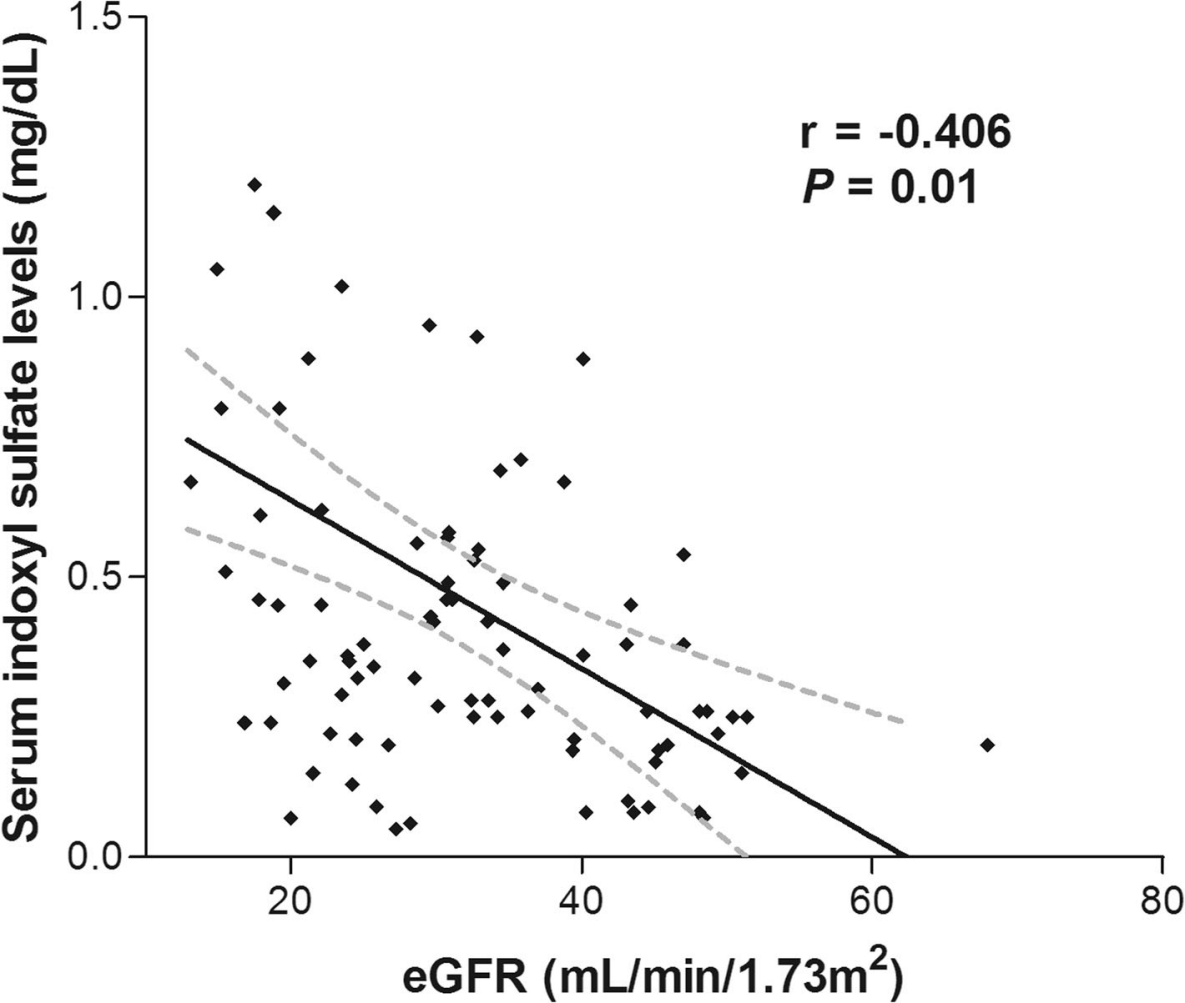
Correlation between indoxyl sulfate and renal function (*r* = coefficient of linear regression). *Abbreviation:* eGFR, estimated glomerular filtration rate

Study participants showed serum IS levels greater by almost ten times when compared to the reference range at baseline. Moreover, serum IS levels increased significantly and progressively with increasing CKD stages [stage 3, 0.21 ± 0.21 (reference); stage 4, 0.54 ± 0.52; stage 5, 1.15 ± 054 mg/dL; *P* for trend *=* 0.001, Fig. 1]. In line with other previous studies, there was a significant negative correlation between serum IS levels and baseline eGFR (r = − 0.406, *P* < 0.001, Fig. 2). Next, we performed linear regression analysis to evaluate the correlation between serum IS levels and clinical parameters (Table 2). In the univariable analysis, serum phosphorus and intact parathyroid hormone (PTH) levels showed positive correlation with serum IS levels. However, baseline eGFR and hemoglobin (Hb) levels were negatively correlated with serum IS levels. In multivariable analysis, only eGFR was independently associated with serum IS levels (coefficient β = − 0.013, *P* = 0.02).

**Table 2:** Association between baseline serum indoxyl sulfate levels and clinical parameters

	Univariate		Multivariable	
	B (95% CI)	***P***	B (95% CI)	***P***
Age (per 1 year increase)	0.003 (−0.004 to 0.01)	0.36	–	
BMI (per 1 kg/m^2^ increase)	− 0.014 (− 0.035 to 0.008)	0.22	–	
SBP (per 1 mmHg increase)	0.004 (− 0.004 to 0.012)	0.31	–	
eGFR (per 1 mL/min/1.73m^2^ increase)	−0.015 (− 0.022 to − 0.008)	< 0.001	−0.013 (− 0.018 to0.001)	0.02
Log-transformed UPCR (per 1 increase)	0.028 (−0.050 to 0.107)	0.46	–	
Hemoglobin (per 1 mg/dL increase)	−0.074 (− 0.123 to − 0.031)	0.001	−0.012 (− 0.059 to 0.034)	0.60
Phosphorus (per 1 mg/dL increase)	0.263 (0.130 to 0.391)	< 0.001	0.085 (−0.053 to 0.224)	0.22
Intact PTH (per 1 mg/dL increase)	0.002 (0.001 to 0.003)	< 0.001	0.001 (0.000 to 0.002)	0.05
serum albumin (per 1 g/dL increase)	−0.024 (− 0.183 to 0.135)	0.77	–	

***Abbreviation***: *BMI* body mass index, *SBP* systolic blood pressure, *eGFR* estimated glomerular filtration rate, *UPCR* urine protein to creatinine ratio, *PTH* parathyroid hormone

### Clinical parameters before and after treatment with OSCA

We compared various clinical parameters before and after treatment with OSCA (Table 3). The body weight, diastolic blood pressure, and hemoglobin level were significantly reduced after OSCA treatment. However, there were no significant differences in the levels of BUN, serum creatinine, and eGFR.

**Table 3:** Clinical parameters before and after treatment with OSCA

	Before treatment (***n*** = 87)	After treatment (***n*** = 87)	***P***
**Body weight (kg)**	71.2 ± 13.9	70.6 ± 13.8	0.03
**Systolic BP (mmHg)**	131.2 ± 11.7	128.3 ± 16.1	0.06
**Diastolic BP (mmHg)**	79.0 ± 11.2	75.1 ± 11.2	0.01
**Hemoglobin (g/dL)**	12.1 ± 2.0	11.9 ± 1.9	0.04
**Phosphorus (mg/dL)**	3.6 ± 0.6	3.7 ± 0.7	0.07
**Uric acid (mg/dL)**	7.1 ± 1.8	7.3 ± 1.8	0.05
**BUN (mg/dL)**	35.2 ± 12.7	36.1 ± 12.7	0.37
**Serum creatinine (mg/dL)**	2.5 ± 0.8	2.6 ± 0.9	0.06
**eGFR by Creatinine (mL/min/1.73m**^**2**^**)**	31.7 ± 11.3	32.0 ± 13.1	0.57
**Serum albumin (g/dL)**	3.9 ± 0.6	3.9 ± 0.5	0.43

The paired t-tests were performed to compare the two groups

***Abbreviation***: *OSCA* oral spherical carbon adsorbent, *BMI* body mass index, *BP* blood pressure, CKD chronic kidney disease, *eGFR* estimated glomerular filtration rate

### OSCA treatment effectively reduces serum indoxyl sulfate levels

**Fig. 3:**
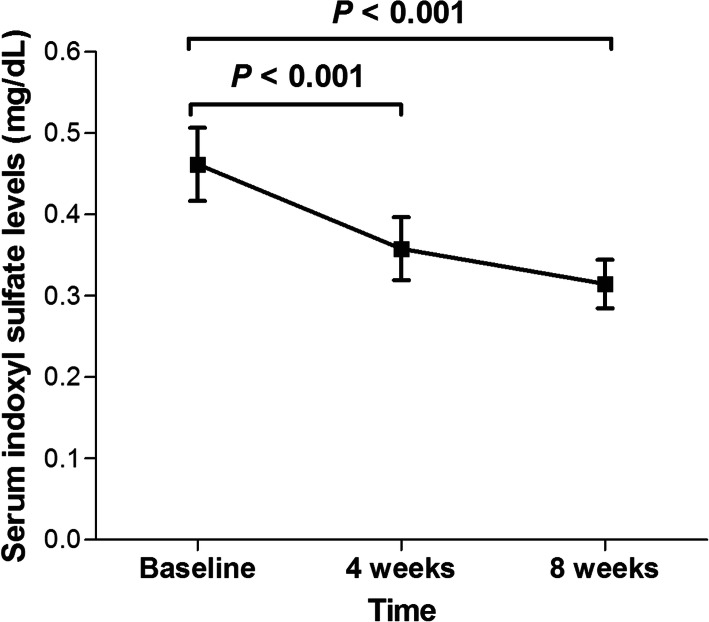
Changes in mean levels of serum indoxyl sulfate from baseline to 8 weeks after OSCA treatment. *Abbreviation:* OSCA, oral spherical carbon adsorbent

**Fig. 4:**
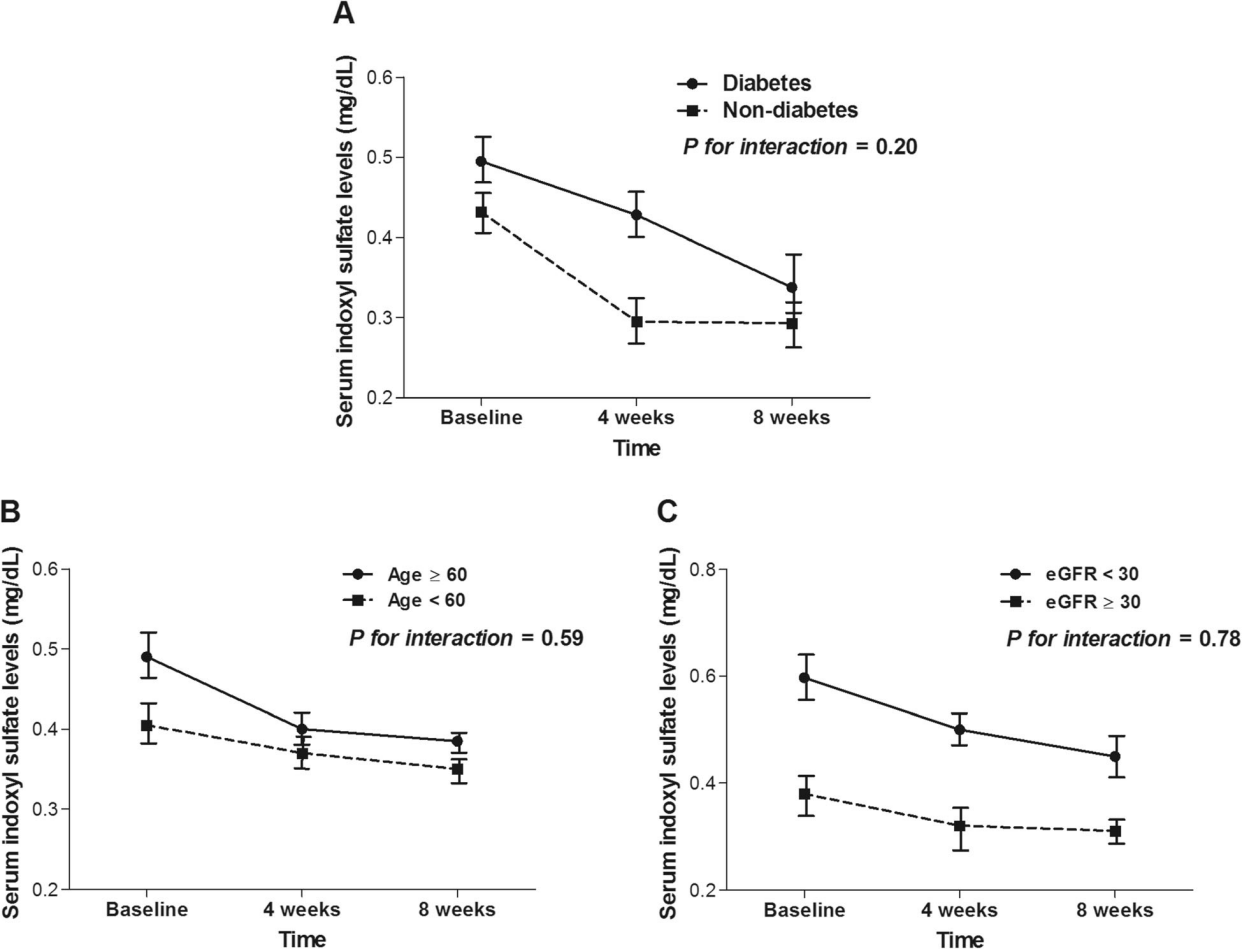
Comparison of reduction in serum indoxyl sulfate by diabetes, age, and baseline renal function. *Abbreviation:* eGFR, estimated glomerular filtration rate

Next, the efficacy of OSCA in reducing serum IS levels was evaluated. Figure 3 shows the mean changes in serum IS level from baseline at 4 and 8 weeks of OSCA treatment for all subjects. OSCA administration significantly reduced serum IS levels at 4 weeks (0.46 ± 0.42 vs. 0.36 ± 0.36 mg/dL, *P* < 0.001) and 8 weeks (0.31 ± 0.28 mg/dL, *P* < 0.001). The degree of reduction in serum IS levels after 4 and 8 weeks of OSCA administration was 22.5 ± 13.9% and 31.9 ± 33.7%, respectively. However, among subjects who were excluded due to failure to take OSCA (*n* = 21), there were no decrease in serum IS levels between baseline and 4 weeks after study enrollment (Additional file 2, Fig. S2). We further performed subgroup analysis, and serum IS levels significantly decreased regardless of age (< 60 vs. ≥60 years), eGFR categories (< 30 vs. ≥30 mL/min/1.73 m^2^), and diabetes (absent vs. present) (Fig. 4). In particular, patients with diabetes showed a significant decrease in serum IS levels at 8 weeks after OSCA treatment (0.49 ± 0.41 vs. 0.33 ± 0.27 mg/dL, *P* < 0.001). Patients without diabetes also showed a significant decrease in serum IS levels at 8 weeks (0.43 ± 0.43 vs. 0.29 ± 0.28, *P* < 0.001).

### Safety and drug compliance

The safety of OSCA was assessed in all enrolled patients who received more than one dose of study medication (*n* = 109) (Table 4). No severe adverse effects requiring admission were reported during the study. The most commonly reported adverse effects were gastrointestinal symptoms (dyspepsia, *n* = 10; constipation, *n* = 3; diarrhea, n = 3; vomiting, *n* = 2) followed by leg cramp (*n* = 1), generalized weakness (*n* = 1), and skin rash (*n* = 1). A total of 22 patients were withdrawn from the study and dyspepsia due to heavy pill burden owing to ingestion of 21 pills per day was the most common reason. The intention-to-treat (ITT) population, including all patients who received at least one dose of the study medication and had at least one post-baseline renal function measurement, was assessed for medication compliance. In the ITT population, the medication compliance rate was 84.7 ± 21.2% for the entire 8 weeks.

**Table 4:** Drug compliance and adverse events

	Total (***n*** = 109)
**Drug compliance**	
First 4 weeks compliance (%)	84.4 ± 21.2
Second 4 weeks compliance (%)	91.6 ± 14.9
Whole period compliance (%)	84.7 ± 21.2
**Adverse events**	
Dyspepsia (%)	10 (47.6)
Diarrhea (%)	3 (14.3)
Constipation (%)	3 (14.3)
Vomiting (%)	2 (9.5)
Skin rash (%)	2 (9.5)
Leg cramps (%)	1 (4.8)
Total adverse effects (n)	21

Data are presented as mean ± standard deviation or number (percentage) of subjects

## Discussion

This study demonstrated that the newly developed carbon adsorbent, OSCA, effectively reduces serum IS levels in moderate to severe CKD patients. The baseline serum IS levels were higher in patients with more advanced stages of CKD. However, at 4 and 8 weeks of OSCA treatment, a significant reduction of serum IS levels from baseline was observed. Furthermore, this reduction effect was independent of age, kidney function, or diabetes. There were no serious side effects of the study medication. The medication compliance rate was 84.7%, and the most common reason for withdrawal was gastrointestinal side effects due to heavy pill burden.

A recent post-hoc subgroup analysis of the EPPIC-USA study per protocol population data suggested that oral absorbent administration might delay the time to primary end point in CKD patients in the USA. A trend of beneficial effects of oral absorbent administration was observed in patients who had study drug compliance rates greater than 67% and were treated for at least 8 weeks [30]. These findings underscore the importance of good patient compliance in achieving benefits of oral adsorbents for preventing the systemic accumulation of uremic toxins. However, in contrast to well-organized clinical studies, many patients report gastrointestinal complications and difficulties in ingesting AST-120 in practice [31].

OSCA is a newly developed oral adsorbent consisting of spherical carbon particles packed in capsules for convenience. An in vitro equilibrium study demonstrated a comparable affinity and capacity constant for indole between OSCA and AST-120. Meanwhile, OSCA demonstrated greater adsorption capacity for β-isobutyric acid and less adsorption capacity for α**-**amylase. These in vitro results suggest an improved selective adsorption capacity of OSCA for indole, which is the main precursor of IS. Such selective adsorption capacity may be due to the improved preservation of the micropore structure by enhanced compression strength. This study is the first clinical study to confirm that OSCA effectively reduces serum levels of IS in moderate to severe CKD patients. OSCA demonstrated up to 31.9% reduction of serum IS levels from baseline, which is comparable to the results of previous clinical studies on AST-120. Such reduction in serum levels of IS was evident at 4 weeks of drug administration. During the course of the present study, there were no significant changes in renal function or blood pressure. Moreover, nutritional parameters, such as serum albumin, also did not show any significant changes during the study period. Thus, it can be speculated that the reduction of serum levels of IS observed in this study is mostly due to OSCA administration.

Another aim of the study was to investigate the adverse effects and medication compliance associated with the study drug in a real clinical setting. The patients were asked to report even the slightest discomfort, and investigators of participating centers were advised to meticulously interview patients to detect adverse effects related to the study drug. No severe treatment-related adverse event was reported. This may have been due to the short duration of the study in contrast to previous EPPIC trials, wherein the median duration of treatment was over 90 weeks. Conversely, OSCA binds to indole in the intestine and is excreted with feces. Thus, it does not accumulate in the body and has less chance to induce toxic effects. Safety results show that the most frequently occurring treatment-emergent and treatment-related adverse events were mainly gastrointestinal disorders. Overall, gastrointestinal symptoms were the most commonly reported treatment-related adverse effect as also seen in Japan and pooled analysis of EPPIC trials [20, 32]. Symptoms, such as dyspepsia, constipation, and diarrhea, accounted for the most reported gastrointestinal symptoms. Most of the symptoms were mild to moderate and none of them required hospitalization. CKD patients belong to the group of patients with chronic disease with the highest daily pill burden [33]. Patients in earlier stages of CKD are reported to take a mean of 6–12 medications that increases depending on the severity of their disease [34]_._ A high pill burden was also prevalent in CKD patients in the present study with more than 4.9 ± 3.6 medications daily. The addition of study medications (21 pills/day) to patients’ daily medications may have contributed to the aggravation of gastrointestinal symptoms and drop-out. Such a high pill burden is usually associated with poor drug compliance. The medication compliance rate was 84.0 ± 23.4% (min 9%, max 101%) in this study over 8 weeks of treatment. This percentage is rather low compared to that in previous clinical trials, which report a compliance rate of over 90%. The EPPIC trials showed high compliance rates and supported the tolerability of oral adsorbent therapy for long-term use. However, whether patients actually took all 30 pills daily cannot be confirmed. Interestingly, our patients showed improved compliance in the second 4 weeks of the study period, which may be attributable to adjustment to the new medication.

The major limitation of this pilot study was that outcomes in the intervention group were not compared with those in a control group. It would have been better to evaluate the efficacy of the study drug in comparison to a placebo. However, at the time of study initiation, OSCA had already been approved for the treatment of progressive CKD in Korea. Therefore, it might not be ethical or safe for patients allocated to the placebo group to ingest so many placebo pills while already having a high pill burden. Moreover, we additionally compared serum IS levels among subjects who were excluded due to failure to take OSCA (*n* = 22). As their compliance for medication was less than 5%, we assessed serum IS levels in these subjects at baseline and at 4 weeks after study enrollment to mimic placebo treated controls. These subjects showed no decrease in serum IS levels between baseline and 4 weeks. Second, Hb levels were decreased among subgroup of subjects after treatment of OSCA. Current Korean National Health Insurance System (KNHIS) allow reimbursement for erythropoietin-stimulating agents (ESAs) therapy in patients with eGFR < 30 mL/min/1.73 m^2^ and Hb levels below 10.0 g/dL. Moreover, the target maintenance Hb range is 10.0 to11.0 g/dL. Therefore, those subjects with eGFR < 30 mL/min/1.73 m^2^ and Hb levels greater than 11.0 g/dL should have stopped their ESAs treatments until Hb levels fell below 10.0 g/dL. In fact, subgroup analysis demonstrated that only subjects with eGFR < 30 mL/min/1.73 m^2^ and Hb levels greater than 11.0 g/dL showed significant decrease in Hb levels during the study. Thus, we speculated that decrease in Hb levels during the study period was the result of reimbursement policy for ESA treatment rather than by the OSCA treatment. Another limitation of this study is the short study duration of 8 weeks. However, Tsubakihara et al. reported that 2 weeks of oral adsorbent therapy can successfully reduce serum levels of IS in advanced CKD patients [23]. Our study also showed that after 4 weeks of OSCA administration, CKD patients showed significant decrease in serum IS levels. Finally, nutritional or dietary compliance was not confirmed by objective methods, such as assessment of 24-h urinary excretion of urea. However, dietary education was provided to all patients and follow-up dietary interviews showed no significant changes in diet in any participant.

## Conclusion

This study demonstrated that a new carbon adsorbent, OSCA, reduced serum levels of IS in moderate to severe CKD patients. In addition, no treatment-related severe adverse effects were reported, and a high medication compliance rate was noted. Future study is warranted to evaluate whether OSCA has further beneficial effects in terms of reduction in the development of renal events in addition to reducing serum levels of uremic toxins.

## Supplementary information

**Additional file 1 Figure S1A and 1B.** Structural features of OSCA.

**Additional file 2 Figure S2.** Changes in serum indoxyl sulfate levels from baseline to 4 weeks after study enrollment among subjects who were excluded due to failure to take OSCA.

## Data Availability

The datasets that support the findings of the current study are available from the corresponding author on reasonable request.

## Abbreviations

CKD: chronic kidney disease
CV: cardiovascular
IS: Indoxyl sulfate
OSCA: Oral spherical carbon adsorbent
ACEi: angiotensin-converting enzyme inhibitors
ARB: angiotensin II receptor blocker
BUN: blood urea nitrogen
UPCR: urine protein to creatinine ratio
PTH: parathyroid hormone
ITT: intention-to-treat
eGFR: Estimated glomerular filtration rate

## References

[1] Levey AS, Atkins R, Coresh J, Cohen EP, Collins AJ, Eckardt KU, Nahas ME, Jaber BL, Jadoul M, Levin A (2007). Chronic kidney disease as a global public health problem: approaches and initiatives - a position statement from kidney disease improving global outcomes. Kidney Int.

[2] Kim KM, Oh HJ, Choi HY, Lee H, Ryu D-R (2019). Impact of chronic kidney disease on mortality: a nationwide cohort study. Kidney Res Clin Pract.

[3] Webster AC, Nagler EV, Morton RL, Masson P (2017). Chronic kidney disease. Lancet.

[4] Sang Jin L, Yoon Chul J, Dong Ok J, Hyo Jin C, Sung Gyu I, Sun Kyung J, Ho Joon K, Mi Jung K, Jang Han L (2013). High serum C-reactive protein level predicts mortality in patients with stage 3 chronic kidney disease or higher and diabetic foot infections. Kidney Res Clin Pract.

[5] Jalal D, Chonchol M, Etgen T, Sander D (2012). C-reactive protein as a predictor of cardiovascular events in elderly patients with chronic kidney disease. J Nephrol.

[6] Perry HM, Okusa MD (2016). Endothelial dysfunction in renal interstitial fibrosis. Nephron.

[7] Goligorsky MS (2015). Pathogenesis of endothelial cell dysfunction in chronic kidney disease: a retrospective and what the future may hold. Kidney Res Clin Pract.

[8] Tsirpanlis G (2008). Cellular senescence, cardiovascular risk, and CKD: a review of established and hypothetical interconnections. Am J Kidney Dis.

[9] Niwa T (2010). Uremic toxicity of indoxyl sulfate. Nagoya J Med Sci.

[10] Vanholder R, De Smet R, Glorieux G, Argiles A, Baurmeister U, Brunet P, Clark W, Cohen G, De Deyn PP, Deppisch R (2003). Review on uremic toxins: classification, concentration, and interindividual variability. Kidney Int.

[11] Aronov PA, Luo FJ, Plummer NS, Quan Z, Holmes S, Hostetter TH, Meyer TW (2011). Colonic contribution to uremic solutes. J Am Soc Nephrol.

[12] Vanholder R, Schepers E, Pletinck A, Nagler EV, Glorieux G (2014). The uremic toxicity of indoxyl sulfate and p-cresyl sulfate: a systematic review. J Am Soc Nephrol.

[13] Barreto FC, Barreto DV, Liabeuf S, Meert N, Glorieux G, Temmar M, Choukroun G, Vanholder R, Massy ZA (2009). Serum indoxyl sulfate is associated with vascular disease and mortality in chronic kidney disease patients. Clin J Am Soc Nephrol.

[14] Sun CY, Hsu HH (2013). Wu MS: p-cresol sulfate and indoxyl sulfate induce similar cellular inflammatory gene expressions in cultured proximal renal tubular cells. Nephrol Dialysis Transpl.

[15] Sun CY, Chang SC, Wu MS (2012). Uremic toxins induce kidney fibrosis by activating intrarenal renin-angiotensin-aldosterone system associated epithelial-to-mesenchymal transition. PLoS One.

[16] Shimizu H, Bolati D, Adijiang A, Muteliefu G, Enomoto A, Nishijima F, Dateki M, Niwa T (2011). NF-kappaB plays an important role in indoxyl sulfate-induced cellular senescence, fibrotic gene expression, and inhibition of proliferation in proximal tubular cells. Am J Physiol Cell Physiol.

[17] Lin CJ, Wu V, Wu PC, Wu CJ (2015). Meta-analysis of the associations of p-Cresyl sulfate (PCS) and Indoxyl sulfate (IS) with cardiovascular events and all-cause mortality in patients with chronic renal failure. PLoS One.

[18] Lekawanvijit S, Kompa AR, Krum H (2016). Protein-bound uremic toxins: a long overlooked culprit in cardiorenal syndrome. Am J Physiol Renal Physiol.

[19] Yang CY, Tarng DC (2018). Diet, gut microbiome and indoxyl sulphate in chronic kidney disease patients. Nephrology (Carlton).

[20] Niwa T, Emoto Y, Maeda K, Uehara Y, Yamada N, Shibata M (1991). Oral sorbent suppresses accumulation of albumin-bound indoxyl sulphate in serum of haemodialysis patients. Nephrol Dialysis Transpl.

[21] Owada A, Nakao M, Koike J, Ujiie K, Tomita K, Shiigai T (1997). Effects of oral adsorbent AST-120 on the progression of chronic renal failure: a randomized controlled study. Kidney Int Suppl.

[22] Niwa T, Nomura T, Sugiyama S, Miyazaki T, Tsukushi S, Tsutsui S (1997). The protein metabolite hypothesis, a model for the progression of renal failure: an oral adsorbent lowers indoxyl sulfate levels in undialyzed uremic patients. Kidney Int Suppl.

[23] Tsubakihara Y, Takabatake Y, Oka K, Shoji T, Togawa M, Okada N, Takahito I, Imai E (2003). Effects of the oral adsorbent AST-120 on tryptophan metabolism in uremic patients. Am J Kidney Dis.

[24] Iida S, Kohno K, Yoshimura J, Ueda S, Usui M, Miyazaki H, Nishida H, Tamaki K, Okuda S (2006). Carbonic-adsorbent AST-120 reduces overload of indoxyl sulfate and the plasma level of TGF-beta1 in patients with chronic renal failure. Clin Exp Nephrol.

[25] Schulman G, Agarwal R, Acharya M, Berl T, Blumenthal S, Kopyt N (2006). A multicenter, randomized, double-blind, placebo-controlled, dose-ranging study of AST-120 (Kremezin) in patients with moderate to severe CKD. Am J Kidney Dis.

[26] Kopple JD (2001). National kidney foundation K/DOQI clinical practice guidelines for nutrition in chronic renal failure. Am J Kidney Dis.

[27] Levey AS, Bosch JP, Lewis JB, Greene T, Rogers N, Roth D (1999). A more accurate method to estimate glomerular filtration rate from serum creatinine: a new prediction equation. Modification of diet in renal disease study group. Ann Intern Med.

[28] Duranton F, Cohen G, De Smet R, Rodriguez M, Jankowski J, Vanholder R, Argiles A (2012). Normal and pathologic concentrations of uremic toxins. J Am Soc Nephrol.

[29] Faul F, Erdfelder E, Lang AG, Buchner A (2007). G*power 3: a flexible statistical power analysis program for the social, behavioral, and biomedical sciences. Behav Res Methods.

[30] Schulman G, Berl T, Beck GJ, Remuzzi G, Ritz E, Shimizu M, Shobu Y, Kikuchi M (2016). The effects of AST-120 on chronic kidney disease progression in the United States of America: a post hoc subgroup analysis of randomized controlled trials. BMC Nephrol.

[31] Aoyama I, Niwa T (2001). Molecular insights into preventive effects of AST-120 on the progression of renal failure. Clin Exp Nephrol.

[32] Schulman G, Berl T, Beck GJ, Remuzzi G, Ritz E, Arita K, Kato A, Shimizu M (2015). Randomized placebo-controlled EPPIC trials of AST-120 in CKD. J Am Soc Nephrol.

[33] Burnier M, Pruijm M, Wuerzner G, Santschi V (2015). Drug adherence in chronic kidney diseases and dialysis. Nephrol Dialysis Transpl.

[34] Bailie GR, Eisele G, Liu L, Roys E, Kiser M, Finkelstein F, Wolfe R, Port F, Burrows-Hudson S, Saran R (2005). Patterns of medication use in the RRI-CKD study: focus on medications with cardiovascular effects. Nephrol Dialysis Transpl.

