# Supplementary material for: New oral spherical carbon adsorbent effectively reduces serum indoxyl sulfate levels in moderate to advanced chronic kidney disease patients: a multicenter, prospective, open-label study
Source: BMC Nephrol. 2020 Jul 31;21:317. doi: 10.1186/s12882-020-01971-x (PMC7394678; doi:10.1186/s12882-020-01971-x)
Supplement: Supplementary file 1 — Additional file 1 Figure S1A and 1B. Structural features of OSCA. [file 12882_2020_1971_MOESM1_ESM.docx]

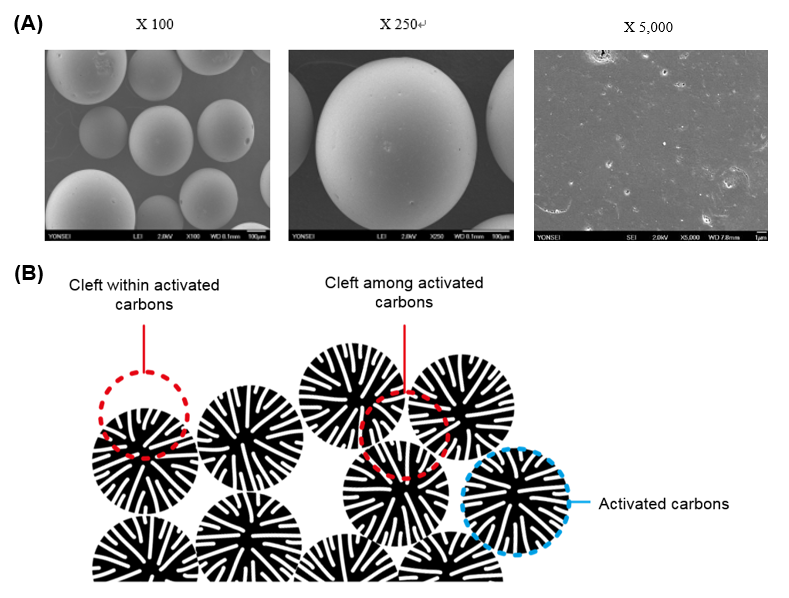


**Additional file 1. Figure S1** Structural features of Renamezin. Scanning electron microscopy of demonstrate that Renamezin presents as black, spherical carbon particles of 0.2-0.4 mm in diameter with hard structural surface (x100, x250, x5,000, **A**). Clefts within activated carbons exhibit increased adsorption to low molecular weight uremic toxins and less adsorption to high molecular weight substances such as digestive enzymes (**B**). (Patent registration number 10-1145131-0000 Puresphere, Spherical activated carbons).

***Note:* (A)** The images were obtained by scanning electronic microscopy by EM-30 (COXEM, Daejeon, South Korea). **(B)** Image was developed by Photoshop software (Adobe Systems, Inc. San Jose, CA, USA).
