# Supplementary material for: New oral spherical carbon adsorbent effectively reduces serum indoxyl sulfate levels in moderate to advanced chronic kidney disease patients: a multicenter, prospective, open-label study
Source: BMC Nephrol. 2020 Jul 31;21:317. doi: 10.1186/s12882-020-01971-x (PMC7394678; doi:10.1186/s12882-020-01971-x)
Supplement: Supplementary file 2 — Additional file 2 Figure S2. Changes in serum indoxyl sulfate levels from baseline to 4 weeks after study enrollment among subjects who were excluded due to failure to take OSCA. [file 12882_2020_1971_MOESM2_ESM.docx]

**
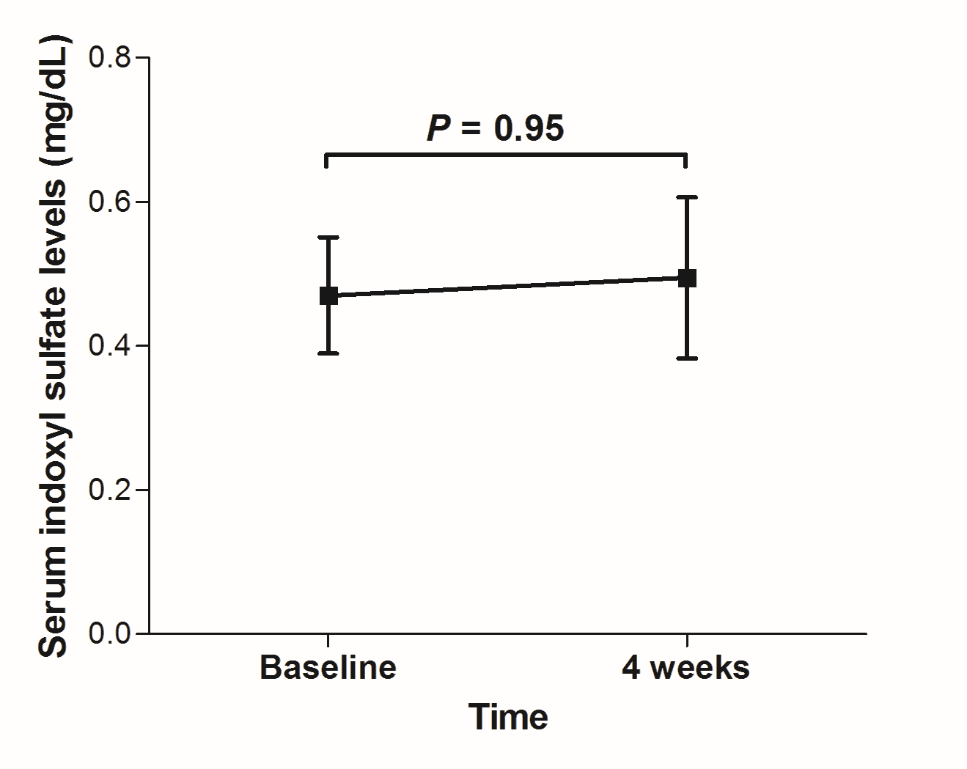
**

**Additional file 2. Figure S2.** Changes in serum indoxyl sulfate levels from baseline to 4 weeks after study enrollment among subjects who were excluded due to failure to take OSCA

***Abbreviation:*** OSCA, oral spherical carbon adsorbent
